# Identifying side effects of commonly used drugs in the treatment of Covid 19

**DOI:** 10.1038/s41598-020-78697-1

**Published:** 2020-12-09

**Authors:** İrfan Aygün, Mehmet Kaya, Reda Alhajj

**Affiliations:** 1 Department of Software Engineering, Celal Bayar University, Manisa, Turkey; 2 Department of Computer Engineering, Fırat University, Elazığ, Turkey; 3 Department of Computer Science, University of Calgary, Calgary, AB Canada; 4 Department of Computer Engineering, Isstanbul Medipol University, Istanbul, Turkey; 5 Department of Health Informatics, University of Southern Denmark, Odense, Denmark

**Keywords:** Information technology, Drug safety

## Abstract

To increase the success in Covid 19 treatment, many drug suggestions are presented, and some clinical studies are shared in the literature. There have been some attempts to use some of these drugs in combination. However, using more than one drug together may cause serious side effects on patients. Therefore, detecting drug-drug interactions of the drugs used will be of great importance in the treatment of Covid 19. In this study, the interactions of 8 drugs used for Covid 19 treatment with 645 different drugs and possible side effects estimates have been produced using Graph Convolutional Networks. As a result of the experiments, it has been found that the hematopoietic system and the cardiovascular system are exposed to more side effects than other organs. Among the focused drugs, Heparin and Atazanavir appear to cause more adverse reactions than other drugs. In addition, as it is known that some of these 8 drugs are used together in Covid-19 treatment, the side effects caused by using these drugs together are shared. With the experimental results obtained, it is aimed to facilitate the selection of the drugs and increase the success of Covid 19 treatment according to the targeted patient.

## Introduction

As of December 2019, a coronavirus species that can spread from person to person was identified in Wuhan, China^1^. The disease later on called Covid-19 posed a risk to be declared as a pandemic by the World Health Organization (WHO) in a short time^2^. As of the date of this study in early June 2020, more than 6.4 million people were infected with this virus, and 373,334 persons died. It has been realized that scientists and researchers have published thousands of clinical trial results and articles in this process to provide treatment methods for the disease^3^. An important part of these studies examines the use of existing drugs for Covid-19 treatment, and suggests possible treatment methods^4–6^.

One of the issues that should be examined before recommending a drug to a patient and after treatment is the side effects of the drug. Research shows that multiple drugs usage (polypharmacy) significantly increases drug side effects^7,8^. For older patients, the probability of polypharmacy generally increases. However, studies clearly show that as the number of drugs used increases, the negative effects seen in patients may also increase^9–11^. Therefore, it is vital to predict drug-drug interactions (DDI) and adverse drug reactions (ADR) for the drugs to be used in the treatment of a disease^8,12^. Knowing the side effects and DDI of the drugs recommended in Covid-19 treatment will play an important role in the success of the process.

According to statistical studies, the vast majority of Covid-19 patients are seen at age 50 and over (Sobotka et al. 2020). According to the results of polypharmacy studies, regular and multiple drugs usage is over 60% in this age group^7^. When these two examinations are evaluated together, it is understood that the rates of multiple drugs usage for Covid-19 patients are quite high. For this reason, the importance of DDI studies increases in Covid-19 treatment.

Today, DDI research efforts are mainly conducted using computer-based methods^13^. In this paper, possible interactions of drugs used in Covid-19 treatment will be examined by employing graph convolutional networks. The aim of the study is to create a projection on the interactions of drugs used in Covid-19 treatment with other drugs. This way, it is aimed to contribute to increasing the success of the treatment by reducing the negative effects of the drugs. To achieve this, answers to the following questions were sought for each of the 8 drugs examined within the scope of the study.

- Which other drugs used in combination with a given drug will have the most dangerous side effects?
- What are the most common side effects as a result of using another drug with a given drug?
- Which disease or organ system are mostly affected by the side effects caused by the given drug?

## Related work

This section concisely reviews previous research efforts described in the literature. Firstly, DDI studies for Covid-19 treatment are investigated. Then, DDI studies, and methods with different approaches were examined.

### DDI studies for the treatment of COVID 19

Drug interactions for different drugs and disease groups were examined during the treatment of the disease. For example, it is recommended that the risks should be evaluated well before using Ritonavir/Lopinavir drugs in patients with kidney transplantation^14^. In addition, the need for guidelines to be prepared on this subject was emphasized. In a different study^15^, possible effects of drugs were investigated according to the heart rhythm graphics of patients. This research shares the side effects of the combination of Hydroxychloroquine and Azithromycin when used together in Covid-19 treatment. In a more comprehensive research, interactions of 4 different drug groups were examined^16^. In addition to these studies, there are websites which are available for online access by universities and pharmaceutical research laboratories (COVID-19 Drug Information, 2020; Medscape Drug Reference Database, 2020; Liverpool COVID-19 Interactions, 2020).

### Computer based DDI studies

Extracting complex relationships from big data has become possible with today's technology and data analysis methods. Since the concept of DDI focuses on the relationships between drugs, many studies have been conducted using data mining methods^20^. In such studies, a corpus with pharmacological data on drugs is usually used. The next step is to apply data mining methods to extract relationships from this corpus^21–23^.

Another method of relationship extraction using information technology employs neural networks^24^. These structures, consisting of nodes and edges, form the basis for studies that can reveal the relationships between drugs when shown by graphs. Many studies have been presented in the literature using this approach^25–27^. In these studies, nodes were interpreted as drugs, and edges were interpreted as interactions between drugs.

There are also DDI studies specific to a group of drugs or to drugs used to treat a specific disease, rather than focusing on the entire drug network. For example, various studies have been conducted on cancer prevention drugs^28,29^; others tackled high blood pressure patients; and some medications used in the treatment^30,31^. In these studies, only the risks and interactions for the examined group are calculated. Therefore, in addition to a general medical corpus, additional data needs may occur with case or disease focus. However, it becomes possible to obtain faster results because it focuses on a certain region instead of the relationships formed on the whole network.

In 2018, a DDI research called Decagon was completed; it predicts the interactions of 645 drugs^32^. The work done by Zitnik et al. is open to development, and its models are shared so that it can be used in different projects. Accordingly, another study (ESP) (Cohen & Widdows, 2017) representing semantic predictions in pharmacological data was combined with Decagon (Burkhardt et al., 2019). The work done by Burkhardt et al. forms a resource for existing DDI researches, thanks to its rapid training and ease of reuse. In this study, in addition to the biomedical data and graph structure presented by Decagon, the infrastructure of the study conducted by Burkhardt is also used.

## Materıal and methods

### Datasets

This study uses data from the study shared by Burkhardt et al. and pre-trained vectors suitable for reuse^33^. The dataset cluster contains the following data from Decagon.

- 964 different polypharmacy side effects derived from a wider side effect dataset^34^, each seen at least 500 times.
- Graph network consisting of 645 drugs and 19,085 protein nodes (4,651,131 drug-drug, 18,596 drug-protein node)
- Graph network hosting protein–protein and drug-protein relationships (total 8,083,300 pieces)

The LitCovid dataset was used to determine the drugs used in Covid-19 treatment. Covid-19 focused articles published in Pubmed are updated daily and added to this dataset. As of the day of the study in Mid June 2020, there are bibliographic format and summaries of about 17,288 studies within the dataset.

### DDI with graph convolutional networks (GCN)

**Figure 1:**
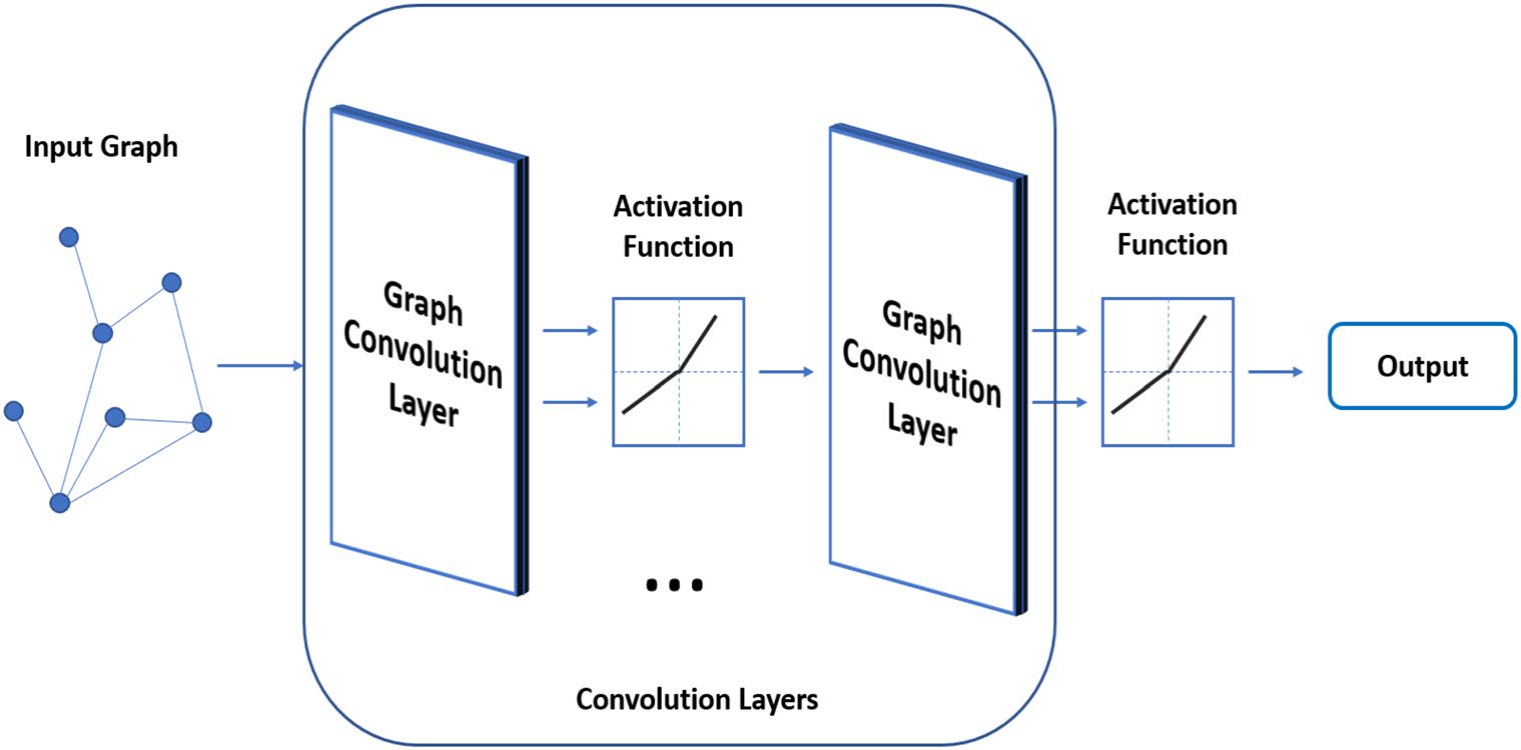
GCN work architecture.

Convolutional Neural Networks (CNN) and GCN are quite similar in architectural structure. However, GCN uses graphs as input^35^. A standard GCN architecture is shown in Fig. 1.

It is aimed to explore graph properties and signals for GCN (Kipf and Welling 2019). It is assumed that each node has the properties it has from neighboring nodes and the relationships it establishes with these nodes (Wu et al. 2019). Due to the Convolution layers and activation function (such as ReLU), the properties of all nodes are scanned. Depending on the study, the GCN output can be produced in different formats as a graph, featuring or representing bilateral relations.

**Figure 2:**
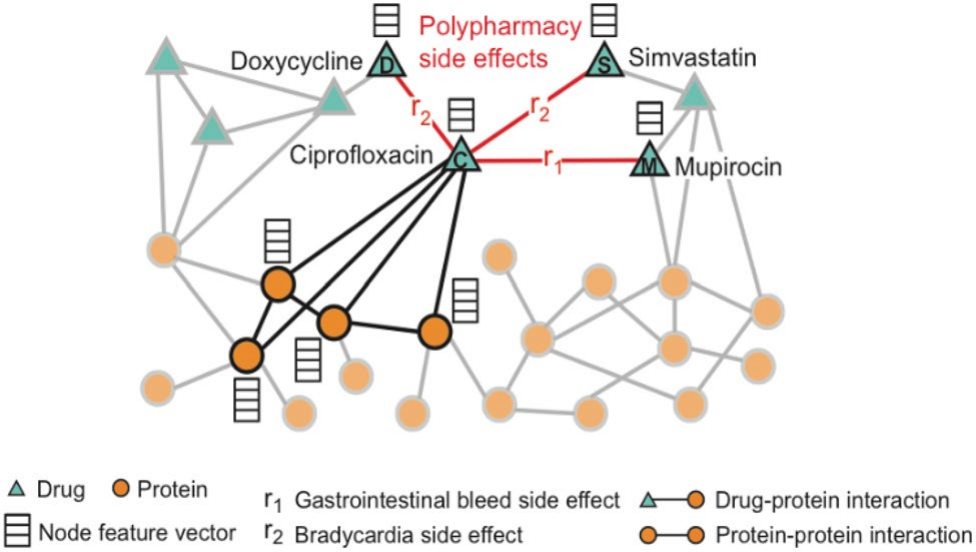
General view of the graph from the Decagon Study, where the nodes and their relationships are defined^32^.

Detecting relationships over biomedical data is one of the main study areas of GCN (Zhang et al. 2019). DDI studies or graph-based studies for the detection of side effects and ADRs are available in the literature. Through these studies, DDI predictions are carried out with different approaches. As shown in Fig. 2, a graph containing drugs and proteins as nodes was created in the Decagon study.

It can be easily realized from Fig. 2 that estimates of possible side effects are produced by examining drug-drug, drug-protein and protein–protein interactions. In this study, the graph shown in Fig. 2 is used as the GCN input and the results of drug interaction (in the form of drug1, drug2, side_effects) are obtained as output. Using this infrastructure, Burkhardt et al. have also classified side effects according to diseases or organ systems^33^.

### Choosing the target drugs

In this study, a combination of three different sources was used to select the drugs whose interactions with other drugs would be calculated. The first is the Decagon study, from which we use the network infrastructure. Another source is the LitCovid dataset, which compiles Covid-19 oriented studies on PubMed. The last one is an online system called "covid19-druginteractions.org" that predicts drug interactions which is shared by Liverpool University.

Firstly, 645 drugs from the Decagon project were chosen in the 'drug_names' dataset. In the next step, the frequency of being in the LitCovid dataset was measured for each of these drugs. The target is to identify the most mentioned drugs in Covid 19 studies published in PubMed. In order to count the number of times the drugs were mentioned, a series of operations were performed in the LitCovid data set. To avoid producing misleading results in the frequency calculation of the terms that are mentioned in the same article, only the abstract sections of the studies were searched. This way, it is easier to get more consistent results regarding the number of papers that include a drug. Data preprocessing steps have been applied to reduce the dataset only to the 'abstract' sections and make it searchable. On the normalized data, 645 drugs were subject to frequency measurement.

After frequency measurement on the LitCovid dataset, it was realized that 543 drugs were never mentioned in the dataset. The mentioning frequency of the remaining 102 drugs in the dataset was calculated as 9.06 on average. However, when we removed the 8 most frequently used drugs from the produced list, the mentioning frequency of the remaining 94 drugs drops below 3. For this reason, experiments continued by focusing only on the first 8 drugs listed in Table 1 with their mentioning frequency in the dataset. Table 1 also shows whether the drugs obtained by the LitCovid scan are available in the online system accessible from the link: www.covid19-druginteractions.org.

**Table 1:** Frequency of drugs according to LitCovid dataset and covid19-druginteractions.org.

Drug name	Drug frequency in LitCovid	Existence in the covid19-druginteractions.org system (Liverpool Uni.)
Hydroxychloroquine	265	✓
Chloroquine	191	✓
Azithromycin	88	✓
Heparin	47	–
Clozapine	24	–
Ritonavir	15	✓
Ribavirin	13	✓
Atazanavir	6	✓

It has been observed that 6 of the selected drugs are also available on the 'http://www.covid19-druginteractions.org' website designed to show drug interactions for Covid-19.

**Figure 3:**
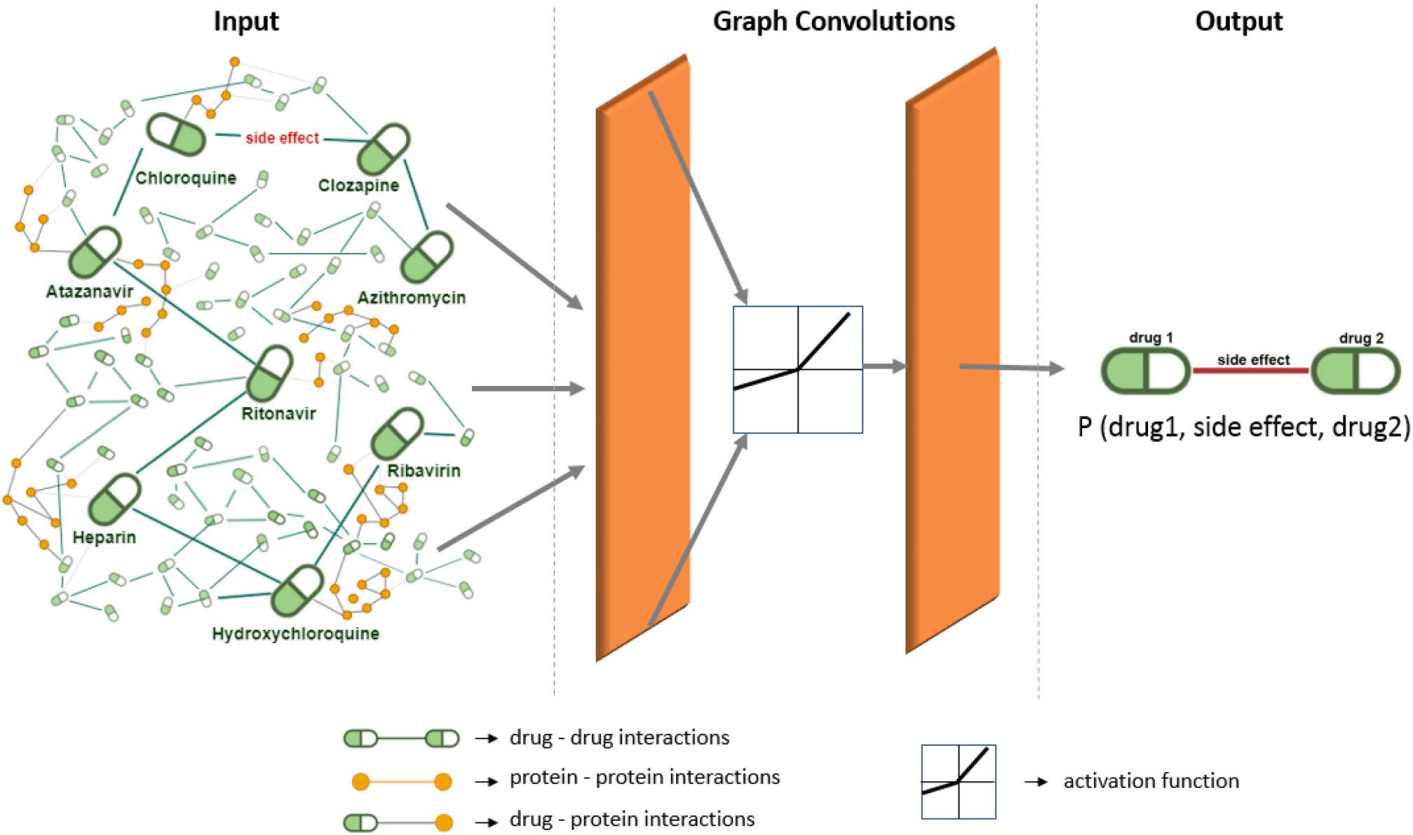
Representation of the GCN architecture and the drug network used in experiments.

Although Heparin and Clozapine drugs are not found in this system, they have been addressed in many studies for Covid-19 treatment^36–39^. Some of the studies on these drugs have been carried out in the form of clinical trials directly on patients with Covid-19. Considering their frequent history in the literature, the two drugs Heparin and Clozapine were included in this study. The drugs to be examined within the scope of the study and the GCN architecture to be applied are shown in Fig. 3 with representative modeling. When Fig. 3 is examined, it can be eassily seen that the drug graph containing 8 focused drugs is used as the GCN input. This network will be analyzed in convolutional steps to generate side effect predictions as output. As can be seen in Fig. 3, the output format to be obtained should consist of triplets in the form (drug1—side effect—drug2).

### Experiments

**Figure 4:**
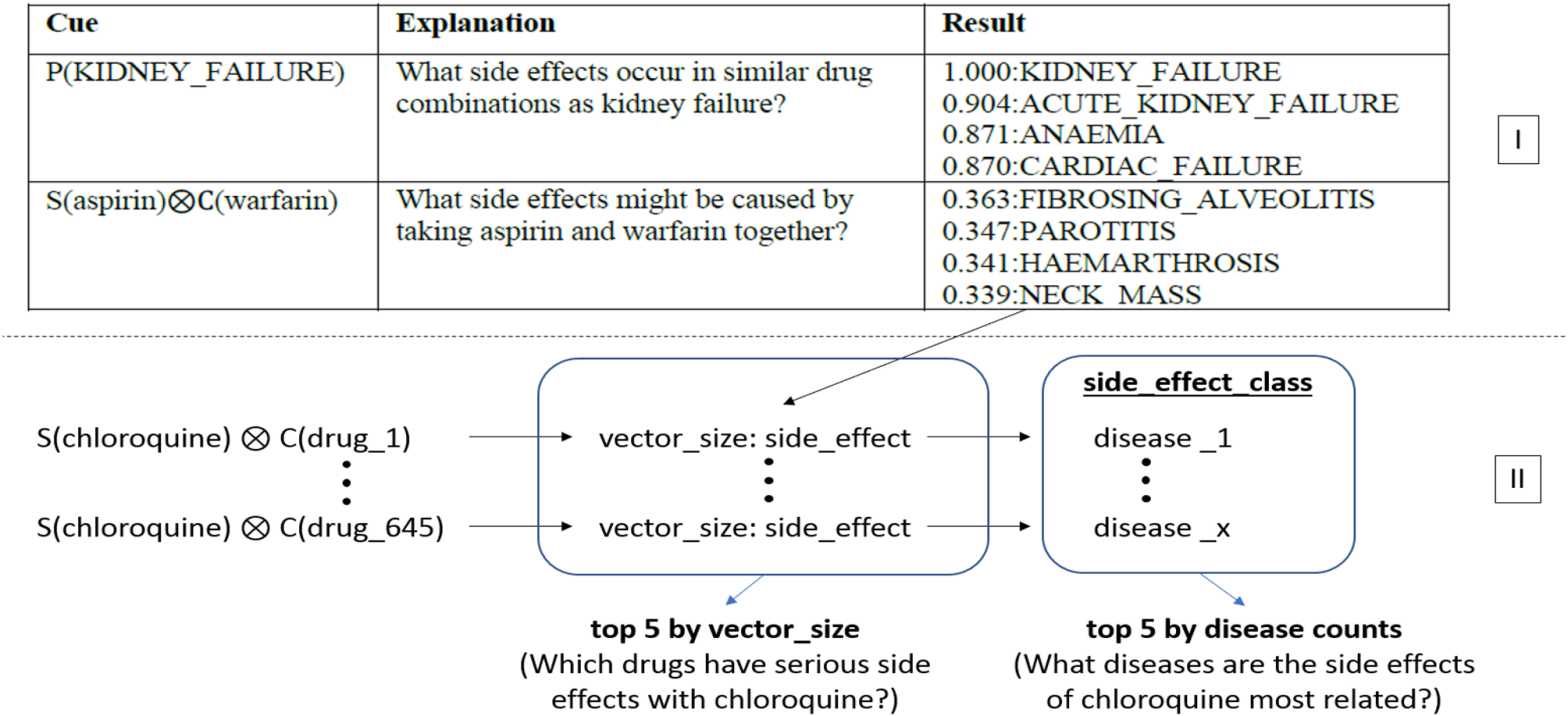
Updates on past studies and showing the logic of the current study with the example of Chloroquine. For Part I^33^.

Experiments were conducted using a modified version of the project shared by Burkhardt, and using the pre-trained vectors of this project^40^. Thus, there was no need for the re-vectorization and training of the Decagon network of medicines and proteins. Only the drugs selected in the previous section were sent as input to the side effect estimation module used in the project. This module was then updated based on the format of each input; interactions with all drugs in the network are monitored and the 5 highest scoring results are produced. It is also possible to measure whether the side effects defined in the project are included in any disease or organ system. In another update, all the side effect results resulting from the interaction of the target drugs with other drugs were classified, and the organ systems and the disease groups that these drugs may cause the most were measured. The way the project works and its differences from previous works is depicted in Fig. 4.

The first part, shown as Section I in Fig. 4, reflects the arthitecture of the past study, while the second part represent the arthitecture of the current study. The vector representation shown in Fig. 4 was created by adding the ESP study to the Decagon study. These vectors are between 0 and 1, indicating that the effect will increase as the value gets closer to 1 and will decrease when it is closer to 0. Throughout the study, all other drugs shown in Table 1 were subject to the same steps, with Chloroquine shown in the example.

## Results and discussion

In this section, the results for each of the drugs are examined under sub-headings. After the interactions of each drug were examined, the resulting side effects were classified. A drug which causes the same side effect as 25 or more drugs will have its side effects are added to the chart. This way, the most common side effects of the 8 drugs used in the experiments have been derived and visualized. Values of the area under the ROC curve (AUROC) and values of the area under precision-re-call curve (AUPRC) were used to measure the performance of the side effect predictions produced in the experiments. The average AUROC value is 0.872 and the average AUPRC value is 0.832 for the types of side effects associated with the Decagon network used in the study. These values are the results of the measurements made separately for 964 different types of side effects. The biomedical literature was consulted in order to present the predictions of the side effects produced due to polypharmacy more strongly. The findings for the drugs studied are presented together with recommendations collected from previous studies specific to these drugs.

**Figure 5:**
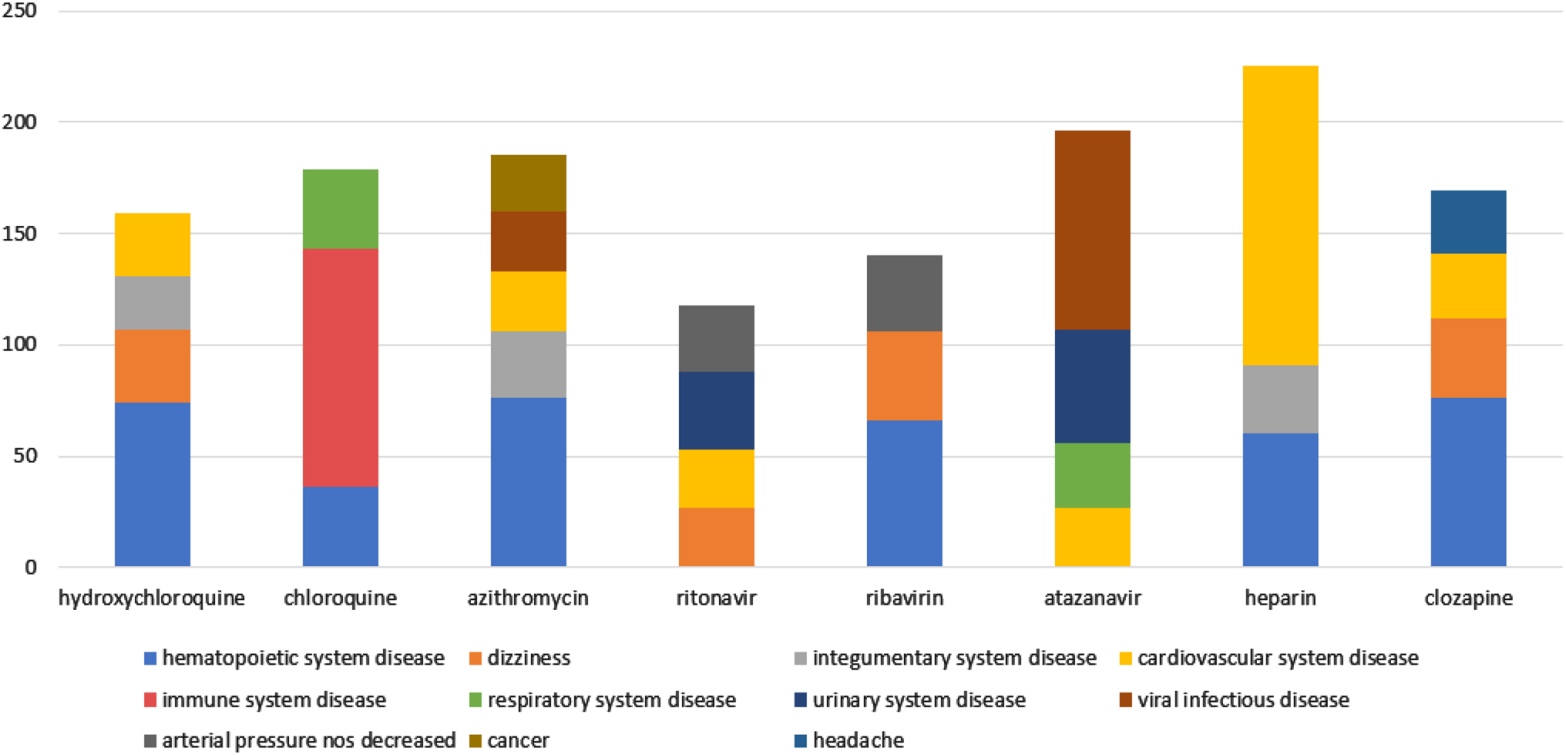
The most common side effects of drugs according to the results of the experiment.

While counting side effects, the rates of the side effects were not taken into consideration. Regardless of these rates, the focus is on which disease group is mostly produced as a result of side effects. However, more detailed data by considering the rates of the side effects are reported in tables given in the sequel. In order to compare the effects of the drugs in general, the chart summarizing the side effects is shown in Fig. 5; it was produced using the 645 drugs in the dataset.

The most common side effects of drugs are seen in Fig. 5. In addition, organ systems the most damaged by these side effects are revealed. Examining the chart, it is possible to infer that Heparin has more cardiovascular system side effects than other drugs, and Chloroquine causes more side effects on the immune system. These are important to create a general drug interaction projection. According to Fig. 4, it can be observed that hematopoietic and cardiovascular system diseases are the most common side effects of drugs. For this reason, patients with a diagnosis of Covid 19 positive and having any of these disease groups will need special attention during the treatment. The reactions of the drugs will be examined in more detail in the following sub-headings.

Following this summary, the rest of this section addresses the following questions for each drug.

- Which are the most dangerous drugs to use in combination with a given drug?
- What are the most common side effects related to the use of other drugs with a given drug?
- Which diseases or organ systems are mostly associated with common side effects?

### Ribavirin

According to the results obtained from the experiments, the drugs which have the highest rate of side effects with Ribavirin are shown in Table 2. The table shows between 2 and 5 side effects for each drug. This variability is because different edges on the graph can show the same side effects.

**Table 2:** The riskiest drugs and possible side effects with Ribavirin concomitant.

Drug name	Side effects that can be seen with ribavirin concomitant	Side effect rates (0–1)
Pregabalin	Abnormal_gait	0.689
	Balance_disorder	0.680
	Aptyalism	0.678
	Diplopia	0.675
Fluticasone	Acute_bronchitis	0.667
	Polyarthritis	0.655
	Bronchitis	0.655
Duloxetine	Anosmia	0.666
	Excessive_sleepiness	0.658
	Abnormal_gait	0.654
Valdecoxib	Arthritis	0.663
	Acid_reflux	0.658
	Osteoarthritis	0.654
Varenicline	Aptyalism	0.654
	Amnesia	0.649
	Insomnia	0.647

From the experiments, the riskiest drugs to be used with Ribavirin are listed in Table 2. Among the 644 other drugs used in the study with Ribavirin, the most common side effects are shown in Table 3. There is an important issue to be considered when examining the data in this table. For the interaction of two drugs, a side effect prediction is always produced. These estimates vary between 0 and 1 with a value closer to 1 increases the likelihood of side effects. All the side effects produced in this study were included in the calculation. For this reason, an alternative display which considers the rates of effects is presented in the second column. In the second part of Table 3, while preparing the side effects column with the highest impact rate, the vectors created for that side effect were averaged. Other tables related to the remaining drugs have been created following the same format.

**Table 3:** Side effects according to the average frequency and effect rate depending on drug use with Ribavirin.

Most common side effects	Number	Side effects with the highest effect rate	Side effect average (0–1)
Thrombocytopenia	42	Balance_disorder	0.680
Dizziness	40	Diplopia	0.675
Arterial_pressure_nos_decreased	34	Acute_bronchitis	0.667
Aspergillosis	27	Anosmia	0.666
Neutropenia	26	Arthritis	0.663

According to Table 3, due to the use of different drugs together with Ribavirin, it is likely that thrombocytopenia and dizziness complaints may be seen. Even though these are not very common in some drug combinations (such as ribavirin-pregabalin), the loss of balance and the possibility of double vision have been calculated to be quite high.

### Chloroquine

The drugs with the highest rate of side effects in use with Chloroquine are listed in Table 4.

**Table 4:** Drugs with the highest rate of side effects when used with Chloroquine.

Drug name	Side effects that can be seen when using with chloroquine	Side effect rates (0–1)
Methotrexate	Candida_infection	0.639
	Lung_neoplasms	0.634
	Adenopathy	0.632
Prednisolone	Peliosis	0.571
	Interstitial_nephritis	0.561
	Bilirubinaemia	0.559
	Agranulocytoses	0.559
Folate	Herpes_zoster	0.555
	Adenocarcinoma	0.550
	Angiitis	0.548
	Skin_lesion	0.548
Omeprazole	Angiitis	0.548
	Lung_neoplasms	0.545
	Night_sweat	0.544
Lisinopril	Bundle_branch_block_left	0.546
	Cardiomyopathy	0.536

When Table 4 is examined, the five drugs Methotrexate, Prednisolone, Folate, Omeprazole and Lisinopril when used together with Chloroquine show the highest rate of ADR. The side effects seen with Methotrexate, which is generally used for cancer patients, are also mostly associated with cancer^41^. These results can be interpreted as reducing the effect of Methotrexate when Chloroquine and Methotrexate drugs are used together. It is possible to establish such connections for other drugs in the table. For instance, 'adenopathy' affects the lymph nodes or 'lung neoplasms' in the lungs, and are often observed in cancer cases^42,43^. Table 5 examines the most common side effects with Chloroquine.

**Table 5:** Side effects according to average frequency and effect rate due to drug use with Chloroquine.

Most common side effects	Number	Side effects with the highest effect rate	Side effect rate (0–1)
Primary_biliary_cirrhosis	107	Candida_infection	0.633
Tracheitis	45	Adenopathy	0.632
Anaemia_hypochromic	36	Lung_neoplasms	0.589
Kidney_transplant	35	Peliosis	0.569
Cervical_vertebral_fracture	27	Interstitial_nephritis	0.561

According to Table 5, Chloroquine has the possibility of showing the side effect defined as 'primary biliary cirrhosis' with one of every 6 drugs in the experiment set. A study shows that this side effect is more effective on women^44^. With further interpretation by experts, it is aimed to increase the success in Covid 19 treatment.

### Ritonavir

Drugs with the highest rate of side effects when used with Ritonavir are listed in Table 6.

**Table 6:** Drugs with the highest rate of side effects when used with Ritonavir.

Drug name	Side effects that can be seen when using with ritonavir	Side effect rates (0–1)
Tenofovir_disoproxil_fumarate	Blood_in_urine	0.717
	Acute_kidney_failure	0.712
	Hyperglycaemia	0.711
Atazanavir	Interstitial_nephritis	0.689
	Allergic_dermatitis	0.683
	Disorder_renal	0.682
	Aspartate_aminotransferase_increase	0.681
tmc114	Kidney_failure	0.657
	Anaemia	0.652
Fosamprenavir	Head_ache	0.654
	Nausea	0.647
	Fatigue	0.641
Valganciclovir	Sepsis	0.652
	Pleural_effusion	0.643
	Thrombocytopenia-2-INV	0.639

Ritonavir has a very high rate of side effects with the drugs listed in Table 6. Among these drugs, special attention should be paid to Atazanavir because it is used in Covid 19 treatment^45^. The side effects that may occur when the two drugs are used together are mentioned in detail later on under the subheading of Atazanavir. Table 7 reports the most common side effects of Ritonavir with other drugs.

**Table 7:** Side effects according to average frequency and effect rate due to drug use with Ritonavir.

Most common side effects	Number	Side effects with the highest effect rate	Side effect rate (0–1)
Renal_tubular_acidosis	35	Blood_in_urine	0.712
Arterial_pressure_nos_decreased	30	Allergic_dermatitis	0.620
Dizziness	27	Dermatitis_medicame	0.589
HIV_disease	27	Ntosa disorder_renal	0.610
Cardiovascular_collapse	26	Bronchitis	0.604

According to Table 7, 'renal tubular acidosis' may be claimed as the most common side effect of Ritonavir. The reason for 'hiv disease' among other popular side effects is the use of Ritonavir in HIV treatment^46^. This result shows that some drugs reduce the effect of Ritonavir (on HIV) and cause it to lose its treatment properties.

### Azithromycin

The drugs with the highest rate of side effects when used with Azithromycin are listed in Table 8.

**Table 8:** Drugs with the highest rate of side effects when used with Azithromycin.

Drug name	Side effects that can be seen when using with azithromycin	Side effect rate (0–1)
Thyroxine	Arthritis_infective	0.643
	Vitamin_D_deficiency	0.628
	Macrocytosis	0.626
	Soft_tissue_injuries	0.625
Bupropion	Appendectomy	0.638
	Hypermetropia	0.637
Naproxen	Arthritis_infective	0.629
	Hypermetropia	0.624
	Appendectomy	0.623
	Blood_pressure_abnormal	0.620
Nicotinic_acid	Cerebral_vascular_disorder	0.622
	Dry_eye	0.618
	Carcinoma_of_the_colon	0.615
Acetaminophen	Fibrosing_alveolitis	0.617
	Anisocoria	0.615
	Bronchiolitis	0.615
	Encephalitis_viral	0.613

According to Table 8, the drugs with the highest rate of side effects in use with Azithromycin are Thyroxin, Bupropion, Naproxen, Nicotinic Acid and Acetaminophen. It is also important to note that Azithromycin and Hydroxychloroquine drugs are used together in Covid 19 treatment^47^. Interactions between these two drugs were reported later on when we talk about Hydroxychloroquine. The most common side effects when using the other 644 drugs with Azithromycin are shown in Table 9.

**Table 9:** Side effects according to average frequency and effect rate due to drug use with Azithromycin.

Most common side effects	Number	Side effects with the highest effect rate	Side effect rate (0–1)
Thrombocytopenia	39	Appendectomy	0.630
Neutropenia	37	Macrocytosis	0.626
Lyell	30	Hypermetropia	0.625
Cardiovascular_collapse	27	Soft_tissue_injuries	0.625
Bone_marrow_failure	25	Cerebral_vascular_disorder	0.622

According to Table 9, the most common side effects with Azithromycin are 'thrombocytopenia' and 'neutropenia'. These two side effects are included in the hematopoietic system byconsidering the classification in the experiments. Based on this ressult, special attention should be paid when using Azithromycin for patients with hematopoietic system disease in Covid 19 treatment.

### Heparin

The drugs with the highest side effects rate when used with heparin are listed in Table 10.

**Table 10:** Drugs with the highest rate of side effects when used with Heparin.

Drug name	Side effects that can be seen when using with heparin	Side effect rate (0–1)
Leucovorin	Leucopenia	0.563
	Hypogammaglobulinaemia	0.548
	Disorder_lung	0.543
	Multifocal_leukoencephalopathy	0.541
Cytosine_arabinoside	Hypogammaglobulinaemia	0.563
	Bilirubinaemia	0.546
	Multifocal_leukoencephalopathy	0.545
Pamidronate	Soft_tissue_infection	0.562
	Fistula	0.555
	Periodontal_disease	0.539
Propofol	Lyell	0.559
	Cerebral_artery_embolism	0.549
	Carcinoma_of_the_colon	0.615
Bupropion	Animal_bite	0.548
	Cluster_headache	0.539
	Defaecation_urgency	0.536
	Mumps	0.532

According to Table 10, the rates of side effects of Heparin do not reach 0.6 in any drug. Among the drugs examined within the scope of the experiments, this ratio stands out as the lowest level. Other drugs with the highest rate of ADR are Leucovorin, Cytosine Arabinoside, Pamidronate, Propofol and Bupropion. Although the rates of side effects with these drugs are low compared to other test drugs, especially the excess number of side effects shown on the cardiovascular system can be clearly seen in the bar shown in Fig. 4 and from the results reported in Table 11.

**Table 11:** Side effects according to average frequency and effect rate due to drug use with heparin.

Most common side effects	Number	Side effects with the highest effect rate	Side effect rate (0–1)
Cardiovascular_collapse	70	Cluster_headache	0.539
Heart_attack	64	Periodontal_disease	0.539
Lyell	31	Hypogammaglobulinaemia	0.537
Neutropenia	30	Dermatitis_exfoliative	0.537
Thrombocytopenia	30	Status_epilepticus-inv	0.533

According to Table 11, Heparin can cause discomfort in the cardiovascular system for one out of every 5 drugs in the test set. The effects of this drug on the cardiovascular system, and side effects such as thrombocytopenia are supported by past studies^48,49^. For this reason, other diseases of the patient should be considered in the process of using Heparin for individual or drug combinations in Covid 19 treatment.

### Hydroxychloroquine

Table 12 reports five drugs with the highest rate of side effects when used together with Hydroxychloroquine.

**Table 12:** Drugs with the highest rate of side effects when used with Hydroxychoroquine.

Drug name	Side effects that can be seen when using with hydroxychloroquine	Side effect rate (0–1)
Rofecoxib	Cerumen_impaction	0.645
	Easy_bruisability	0.633
	Spondylitis	0.633
	Soft_tissue_injuries	0.625
Salbutamol	Cholecystitis_acute	0.631
	Atrial_septal_defect	0.618
	Endocrine_disorder-2	0.616
Quetiapine	Nephrogenic_diabetes_insipidus	0.627
	Schizoaffective_disorder	0.623
	Psychosexual_disorder	0.620
Acetaminophen	Serum_sickness	0.623
	Duodenal_ulcer_perforation	0.622
	Hypogammaglobulinaemia	0.620
	Nodule_skin	0.619
Azithromycin	Hyperlipaemia	0.620
	Nephrosclerosis	0.616
	Hernia	0.606

Particular attention should be paid to the use of Hydroxychloroquine with Azithromycin since both drugs may be used in Covid 19 treatment. In case of using these drugs together, side effects such as 'hyperlipidaemia' and 'nephrosclerosis' may occur. Previous studies showed that both side effects cause hypertension^50,51^. Concomitant use of these drugs can be considered dangerous in patients at risk of hypertension. This contributes to increase the success in Covid 19 treatment in line with the main goal of the study.

Table 13 reports the most common side effects related to the use of Hydroxychloroquine. According to the experimental results shown in Table 13, one of the most common side effects related to the use of Hydroxychloroquine is 'Thrombocytopenia'. This result is also supported by past studies^52^. It may also cause dizziness due to its use with 33 different drugs.

**Table 13:** Side effects according to average frequency and effect rate due to drug use with Hydroxychloroquine.

Most common side effects	Number	Side effects with the highest effect rate	Side effect rate (0–1)
Thrombocytopenia	42	Easy_bruisability	0.633
Dizziness	33	Hyperlipaemia	0.620
Neutropenia	32	Nodule_skin	0.619
Cardiovascular_collapse	28	Erysipelas	0.615
Lyell	24	Scleroderma	0.611

### Atazanavir

Table 14 shares five drugs with the highest rate of side effects when used with Atazanavir.

**Table 14:** Drugs with the highest rate of side effects when used with Atazanavir.

Drug name	Side effects that can be seen when using with atazanavir	Side effect rate (0–1)
Lamivudine	Dermatitis_medicamentosa	0.642
	Hepatitis_toxic	0.641
	Lymphoma	0.640
	Peliosis	0.636
Ritonavir	Lymphoma	0.589
	Nephrotic_syndrome	0.589
	Dermatitis_medicamentosa	0.588
	Peliosis	0.584
Efavirenz	Dermatitis_medicamentosa	0.571
	Nephrotic_syndrome	0.571
	Lymphoma	0.570
	Disease_of_liver	0.567
Indinavir	Nodule	0.570
	Herpes_simplex	0.567
	Hive	0.567
Didanosine	Disease_of_liver	0.556
	Dermatitis_medicamentosa	0.556
	Nephrotic_syndrome	0.555

Among these drugs, Ritonavir, which is in the second place, draw attention. Because this drug is similarly used in Covid 19 treatment. For this reason, the side effects of the two drugs together should be carefully examined. In addition to the side effects seen in these drugs, Table 15 presents the most common side effects related to Atazanavir use.

**Table 15:** Side effects according to the average frequency and effect rate of drug use associated with Atazanavir.

Most common side effects	Number	Side effects with the highest effect rate	Side effect rate (0–1)
HIV_disease	89	Hepatitis_toxic	0.575
Renal_tubular_acidosis	51	Cyst	0.573
Cryptococcosis	47	Nodule	0.569
Pneumocystis_carinii_pneumonia	29	Disease_of_liver	0.567
Cardiovascular_collapse	27	Hive	0.567

According to the experiments, the most common side effect of Atazanavir due to multiple drug usage is 'hiv_disease'. Frequent occurrence of this side effect can be interpreted as the effect of Atazanavir is restricted by 89 different drugs. Also, Atazanavir is known as a drug used in HIV treatment^53^. When these results are evaluated, it can be concluded that many drugs can suppress the effect of Atazanavir in Corona 19 treatment. Further, 'toxic hepatitis' is seen in the first place among the ADRs seen with the highest effect rate in Table 15. In previous studies, the increase of bilirubin value due to the use of Atazanavir singularly or with Ritonavir were attributed to toxic hepatitis^54^. Although the other values remained constant in the serum analysis, the change of this value was observed. With the anticipation of such side effects, success is expected to increase in Covid 19 treatment.

### Clozapine

As a result of the experiments, the drug estimated to produce the highest rate of side effects with Clozapine are shared in Table 16.

**Table 16:** Drugs with the highest rate of side effects when used with Clozapine.

Drug name	Side effects that can be seen when using with clozapine	Side effect rate (0–1)
Risperidone	Psychoses	0.613
	Excessive_thirst	0.611
	Narcolepsy	0.599
Bupropion	Birth_defect	0.606
	Bleeding_gums	0.596
Thyroxine	Bleeding_gums	0.605
	Nodule_skin	0.601
	Hydronephrosis	0.600
	Lung_neoplasms	0.597
Quetiapine	Excessive_thirst	0.601
	Psychoses	0.595
	Alcohol_consumption	0.594
Acetaminophen	Hydronephrosis	0.601
	Multifocal_leukoencephalopathy	0.598
	Nodule_skin	0.598

The excessive psychological effects seen with the use of these drugs necessitate paying more attention to the usage of this drug. This is because Clozapine is used in the treatment of schizophrenia or similar psychological disorders^55^. Therefore, it is possible to conclude that some drugs suppress Clozapine's treatment effect. The most common side effects and rates with other drugs are presented in Table 17.

**Table 17:** Side effects according to average frequency and effect rate due to drug use with Clozapine.

Most common side effects	Number	Side effects with the highest effect rate	Side effect rate (0–1)
Thrombocytopenia	44	Excessive_thirst	0.606
Dizziness	36	Nodule_skin	0.599
Neutropenia	32	Narcolepsy	0.599
Cardiovascular_collapse	29	Hydronephrosis	0.598
Head_ache	28	Lung_neoplasms	0.597

According to Table 17, 'thrombocytopenia' is the most common side effect associated with polypharmacy together with Clozapine. These results have been confirmed by past studies which have been reported that other side effects such as 'neutropenia' are also common with the drug^56^. Within the scope of these results, the effects of Clozapine use for therapeutic purposes for Covid 19 on the psychological and hematopoietic systems should be taken into consideration. It has also been observed that the drug often causes side effects such as dizziness and headache.

## Conclusion

According to the conducted experiments, DDI estimates were produced for 8 different drugs known to be used in Covid 19 treatment. In this process, the infrastructures and results of past studies have been used^32,33^. Within the scope of the study, in order to be able to perform drug treatment according to the patient, the systems and diseases on which each drug has the most side effects have been identified. A projection is presented to create alternative drugs or methods in the treatment of patients with these diseases or at risk. In addition, other drugs with the highest probability of side effects were calculated. As a result of the conducted research, it has been seen that the hematopoietic system is the most vulnerable organ system against DDI with the examined drugs. The second most affected system is the cardiovascular system. It has been determined that both organ systems have been exposed to serious side effects from 6 different drugs. According to the findings obtained, dizziness, headache and thrombocytopenia are the most common diseases due to multiple use of drugs. Heparin had the highest number of adverse reactions among the examined 8 drugs. Ritonavir, on the other hand, was the drug that had negative interactions with the fewest drugs. In addition, it is estimated that serious side effects may occur if Atazanavir and Ritonavir are used together.

With the findings of this research, it is aimed to contribute to Covid 19 treatment of patients who are in regular medication use. It is very important to determine if there is a possibility that the new drug will react negatively with the drugs used regularly by the patient to treat his/her other diseases. For this reason, five different drugs that produce the highest negative interaction score with each drug and their possible side effects were shared in the study. Based on the literature, negative drug interactions of the drugs known to be used together in Covid 19 treatment were also noted. With the outputs obtained as a result of the experiments, suggestions that will contribute to the choices of the drug treatment of experts against Covid 19 and facilitate their choices are presented.

In the study, five possible side effects were calculated for each drug to be used with the other drugs. As a result of these procedures, 25.800 possible side effects occurred for 8 drugs. Since it is not possible to share this much data within the scope of the article, only the first 5 drugs with the highest side effect rate are reported. For this reason, it is planned to transfer the study to the web environment that can show all results. Another possible study in the future will be a web-based project that will list the negative effects of the two drugs selected and indicate their rates. Similarly, a web infrastructure that controls the suitability of the drugs used for Covid 19 can be developed according to the current diseases and the drugs used by the patient. Increasing the dataset to more than 645 drugs will increase the contribution to the literature. Therefore, adding new drugs and proteins to the graph used in the current study is important in order to create an infrastructure that can be used in many DDI studies.
